# Validity of an algorithm for determining sleep/wake states using a new actigraph

**DOI:** 10.1186/1880-6805-33-31

**Published:** 2014-10-04

**Authors:** Kyoko Nakazaki, Shingo Kitamura, Yuki Motomura, Akiko Hida, Yuichi Kamei, Naoki Miura, Kazuo Mishima

**Affiliations:** Department of Psychophysiology, National Institute of Mental Health, National Center of Neurology and Psychiatry, 4-1-1 Ogawa-higashi, Kodaira, Tokyo, 187-8553 Japan; National Center Hospital, National Center of Neurology and Psychiatry, 4-1-1 Ogawa-higashi, Kodaira, Tokyo, 187-8551 Japan; Structural Materials Sector, Materials Science Research Laboratory, Central Research Institute of Electric Power Industry, 2-6-1 Nagasaka, Yokosuka, Kanagawa, 240-0196 Japan

**Keywords:** polysomnography, actigraphy, sleep/wake scoring algorithm, validation, sleep estimation

## Abstract

**Background:**

This study aimed to develop an algorithm for determining sleep/wake states by using chronological data on the amount of physical activity (activity intensity) measured with the FS-750 actigraph, a device that can be worn at the waist, allows for its data to be downloaded at home, and is suitable for use in both sleep research and remote sleep medicine.

**Methods:**

Participants were 34 healthy young adults randomly assigned to two groups, A (n =17) and B (n =17), who underwent an 8-hour polysomnography (PSG) in the laboratory environment. Simultaneous activity data were obtained using the FS-750 attached at the front waist. Sleep/wake state and activity intensity were calculated every 2 minutes (1 epoch). To determine the central epoch of the sleep/wake states (*x*), a five-variable linear model was developed using the activity intensity of Group A for five epochs (*x*_-2_, *x*_-1_, *x*, *x*_+1_, *x*_+2_; 10 minutes). The optimal coefficients were calculated using discriminant analysis. The agreement rate of the developed algorithm was then retested with Group B, and its validity was examined.

**Results:**

The overall agreement rates for group A and group B calculated using the sleep/wake score algorithm developed were 84.7% and 85.4%, respectively. Mean sensitivity (agreement rate for sleep state) was 88.3% and 90.0% and mean specificity (agreement rate for wakeful state) was 66.0% and 64.9%, respectively. These results confirmed comparable agreement rates between the two groups. Furthermore, when applying an estimation rule developed for the sleep parameters measured by the FS-750, no differences were found in the average values between the calculated scores and PSG results, and we also observed a correlation between the two sets of results. Thus, the validity of these evaluation indices based on measurements from the FS-750 is confirmed.

**Conclusions:**

The developed algorithm could determine sleep/wake states from activity intensity data obtained with the FS-750 with sensitivity and specificity equivalent to that determined with conventional actigraphs. The FS-750, which is smaller, less expensive, and able to take measurements over longer periods than conventional devices, is a promising tool for advancing sleep studies at home and in remote sleep medicine.

## Background

Polysomnography (PSG) is regarded as the gold standard in the objective evaluation of sleep/wake states. On the one hand, PSG can distinguish between sleep and wake states and determine the depth of sleep with high accuracy, while on the other hand, evaluating sleep in natural settings with it is difficult because people are mainly evaluated in a laboratory setting while wearing surface electrodes to monitor their brain waves. In addition, investigations for diagnosing circadian rhythm sleep disorder or to ascertain an individual’s sleep habits require continuous monitoring of sleep/wake patterns over the course of a few weeks or months, which is not possible with PSG.

Methods other than PSG for monitoring sleep/wake states include both subjective and objective measurement methods. Although subjective measurement methods such as self-kept sleep diaries are easy to administer, they are less accurate than objective measurement methods since record keeping is retrospective and sleep state misperceptions are possible. The objective method of actigraphy has been attracting recent attention as it can make continuous recordings over long periods in natural settings and places little burden on the subject. Actigraphy uses an actigraph, a lightweight device that can be attached to the wrist or waist, to continuously monitor the amount of physical activity (activity intensity) of the subject. This device can determine the sleep/wake states in epochs of a few seconds to a few minutes by processing the activity data using a unique algorithm. It is reported that the agreement rate between PSG and actigraphy for sleep/wake state determination is in the range of 85% to 96% [1–6]. Actigraphy is a useful method for evaluating an individual’s sleep/wake pattern over the long term, and it has been widely used in clinical studies targeting patients with insomnia or circadian rhythm sleep disorder, as well as in research studies targeting healthy general populations including children [7–10]. Although actigraphy is less expensive compared to PSG, the equipment it requires is still relatively expensive, which has prevented it from being introduced in epidemiological studies targeting large numbers of participants and from being widely used in clinical practice. Its use is also restricted by the fact that a specific interface is needed to download the device’s recorded data, which requires participants to regularly visit a specific research facility or facility specializing in sleep medicine. It is expected that solving these issues will promote its use in large-scale studies and also in remote sleep medicine, and consequently expand the scope of use of actigraphy in research and clinical settings.

The FS-750, distributed recently by Estera Corporation, is an inexpensive device that is worn less obtrusively at the waist than other actigraphs worn at the wrist. It also produces fewer artifacts from movements limited to the upper extremities. Another plus is that the FS-750 can determine the subject’s posture (that is, standing, inverted, supine, lateral (left and right), and prone) from information about the direction the device is facing, which means it can be applied for sleep/wake state determination. The memory capacity is 40 days and it functions for about 6 months with commercially available batteries (CR2032). It uses near field communication (NFC) technology to transmit data and can download its data to a PC using a commercially available NFC reader/writer device.

In this study, to determine whether the FS-750 can be used for monitoring sleep/wake states, we developed an algorithm that estimates sleep/wake states using data on the amount of physical activity (activity intensity) measured with the FS-750 and tested its validity. In addition, following previous studies [2, 11], we developed an estimation rule to optimize the scoring of sleep parameters by the FS-750 and then examined its effectiveness.

## Methods

### Subjects

Forty-one young adults (31 men, 10 women; mean age 22.0 ± 1.8 years) participated in this study. Questionnaire and medical examination results confirmed that the participants had no serious mental, physical, or sleep disorders, were not working nightshifts constantly, had not traveled to a destination with a >6-hour time difference within the 3 months prior to the study, and were not taking medications that might affect the experimental results. To avoid the possibility that abnormal movements of the extremities or trunk would affect the estimation of sleep/wake (S/W) states, data from participants with a periodic limb movement index (PLMI) score of ≥5 on PSG (five participants) and those with an apnea and hypopnea index (AHI) score >15/hour (one participant) were excluded. In addition, data from a participant who had extreme difficulty falling asleep (sleep latency: 207 min) was excluded from the analysis. This left 34 participants (25 men, 9 women; mean age 21.9 ± 1.7 years) as the targets for analysis, and they were randomly assigned to Group A (17 participants) and Group B (17 participants).

This study was approved by the Ethics Committee of the National Center of Neurology and Psychiatry, and written consent was obtained from all participants.

### Polysomnography

The study was conducted in the sleep laboratory unit of the National Institute of Mental Health, National Center of Neurology and Psychiatry.

The participants arrived at 19:00 and after PSG electrodes and the FS-750 were attached, they went to bed at 24:00. Wake-up time was set at 8:00 (maximum 8 hours of sleep), and the participants were directed that if they woke earlier than 8:00 they should not get up and should try to get as much sleep as possible until the lights were turned on. Inside the unit, the temperature was maintained at 25°C and humidity at 50% relative humidity (RH).

PSG recording was made using a Neurofax digital EEG system (EEG-1200, Nihon Koden, Tokyo, Japan) and included an electroencephalogram (EEG) with a conventional montage (F_3_, F_4_, C_3_, C_4_, O_1_, O_2_) based on the contralateral mastoid (A_1_, A_2_), an electrooculogram (EOG) at the outer canthus of each eye, a submental electromyogram (EMG), an electrocardiogram (ECG), an EMG at the left and right tibialis anterior muscles, and monitoring of respiratory signals (oronasal airflow, movements of the chest wall and abdomen, and O_2_ saturation of arterial blood). During the recordings, EEG, EOG, EMG, and ECG signals were digitized at 200 Hz, and the signal was filtered using a high-pass filter with the following time constants: EEG 0.3 s, EOG 0.03 s, submental EMG 0.03 s, and ECG 1.0 s. The signal was filtered using a low-pass filter with the following data: EEG 60 Hz, EOG 60 Hz, submental EMG 60 Hz, and ECG 60 Hz.

EEG recordings from a monopolar lead at C_3_ were assessed visually in 30-second epochs according to the method of Rechtschaffen and Kales [12] and categorized into one of the following sleep stages: Stage Wake, Stage REM, Stage 1, Stage 2, Stage 3, or Stage 4. Four consecutively judged sleep stages (1 epoch =30 seconds) were reclassified as sleep (sleep epochs determined by PSG, S_PSG_ ) or wake (wake epochs determined by PSG, W_PSG_) state every 2 minutes so they corresponded with the activity intensity data measured by the FS-750 (1 epoch =2 minutes). In accordance with a previous study [5], if there were two or more occurrences of Stage Wake in four continuous epochs, this was defined as W_PSG_; otherwise, S_PSG._ S_PSG_ was subdivided into Stage REM, Stage 1, Stage 2, Stage 3, and Stage 4 according to the sleep stage that was most frequent in the four consecutive epochs. However, for epochs that contained different sleep stages occurring at the same frequency, this was categorized according to the order of Stage REM, Stage 1, Stage 2, Stage 3, and Stage 4. (For example, if there were two occurrences of Stage REM and two of Stage 1, S_PSG_ was sub-categorized as Stage REM.)

### Activity recording with the FS-750

Activity during the night while the lights were off was recorded with the FS-750 (Estera Corporation, Saitama, Japan), which is worn at the waist. This small and light, rectangular device (external dimensions: 75 × 33.5 × 10.8 mm (width × height × depth); weight, 26 g including the battery) records the amount of activity by using an internal three-axis accelerometer (electrostatic capacity sensor). Every 0.125 seconds, the number of times that acceleration exceeded a reference value was summed, and the value was recorded as the activity value over 2-minute bins. The activity intensity is calculated from the activity value as a value from 0 to 31 (32 levels). An activity intensity of 0 means the subject did not move, and larger values indicate higher amounts of activities.

### Formulation of an algorithm for sleep/wake scoring

To develop an algorithm for the FS-750 that determines the S/W states, following previous studies [5], a five-dimensional linear model was hypothesized that utilizes activity intensity at an evaluation epoch as well as two epochs before and two epochs after (total 10 minutes). Using the activity intensity at 4 minutes and 2 minutes before the evaluation epoch, at the evaluation epoch, and at 2 minutes and 4 minutes after the epoch (*x*_-2_, *x*_-1_, *x*, *x*_+1_, *x*_+2_), each with a weighting coefficient (*a*_-2_, *a*_-1_, *α*, *a*_+1_, *a*_+2_), the following equation gives composite variable z, which is the discriminant score:


Here, S_PSG_ (=0) and W_PSG_ (=1), which are obtained by PSG (one epoch =2 minutes), are taken as a baseline. Score z classifies the activity intensity obtained from the corresponding epoch by the FS-750 into sleep (S_ACT_) and wake (W_ACT_). The above equation was obtained by discriminant analysis using the data set containing activity intensity data and PSG data (total 4080 epochs) for the 17 participants in Group A. Because data during the 4 minutes before and after the evaluation epoch were needed for the development of an algorithm, activity intensity data staring 4 minutes before and ending 4 minutes after the PSG recording were used in the study.

### Sleep/wake agreement rate, sensitivity, specificity

Using the S/W scoring algorithm developed, the overall agreement rate, sensitivity, and specificity were calculated for the entire recording period as well as for each sleep stage (Stage Wake, Stage REM, Stage 1, Stage 2, and Stage 3 + 4) for each participant in both groups. The overall agreement rate indicates how closely each and all epoch determinations (S_PSG_, W_PSG_) by PSG match the activity intensity score (S_ACT_, W_ACT_) for each corresponding epoch. The agreement rate for each sleep stage determined by PSG (Stage Wake, Stage REM, Stage 1, Stage 2, and Stage 3 + 4) is the percentage of how closely the activity intensity score (S_ACT_, W_ACT_) matches that calculated for each sleep stage. Sensitivity is the ratio of S_ACT_ to S_PSG_ during the entire recording period. Specificity is the ratio of W_ACT_ to W_PSG_ during the entire recording period.

### Optimizing the definitions of sleep parameters

Sleep latency (SL), total sleep time (TST), wake after sleep onset (WASO), and sleep efficiency (SE) were calculated using the S/W data obtained from PSG and activity intensity data for each 2-min epoch [13]. Calculation was performed using the S/W data obtained during time in bed (TIB), where TIB was defined as the recording period, starting at 0:00 when the lights were turned off and ending at 8:00 when the lights were turned on next morning.

The definitions of sleep parameters calculated from the PSG data are as follow:

SL_PSG_ - The interval between the time lights were turned off and the time of the first epoch when any of the sleep stages appeared (sleep-onset time).

TST_PSG_ - The total period of time when sleep (S_PSG_) appeared, from the time of sleep-onset to the time when lights were turned on.

WASO_PSG_ - The time in bed (TIB) from which SL_PSG_ and TST_PSG_ were subtracted.

SE_PSG_ - The ratio of TST_PSG_ to TIB.

The definitions of sleep parameters calculated from the activity intensity data are as follow:

SL_ACT_ - The interval between the time lights were turned off and the time of the first S_ACT_ (sleep-onset time) among the sleep states that appeared continuously for more than *n* epoch for the first time after the lights were turned off. *n* ranged from 1 to 15 (2 to 30 minutes), and SL_ACT_ was calculated for each. When S_ACT_ appeared continuously for more than *n* epochs for the first time after the lights were turned off, the epochs between lights-out and the first S_ACT_ were defined as W_ACT_.

WASO_ACT_ - The total time of W_ACT_ that appeared continuously for more than *n* epochs after sleep onset. *n* ranged from1 to 10 (2 to 20 minutes), and WASO_ACT_ was calculated for each. When W_ACT_ appeared continuously for more than *n* epochs, the epochs were defined as W_ACT_.

TST_ACT_ - The amount of time in TIB from which SL_ACT_ and WASO_ACT_ were substracted.

SE_ACT_ - The ratio of TST_ACT_ to TIB.

For the calculation of SL_ACT_ and WASO_ACT_, the values obtained from the criteria applied above to the values of SL_PSG_ and WASO_PSG_ were compared, and the epoch numbers that would optimize the calculated results were sought. The optimization rules were to minimize the difference between average parameter values obtained by PSG for the 34 participants and the average parameter values obtained from the S/W algorithm so that the difference was not significant, and the intraclass correlation coefficient (ICC) was considered significant when ICC was ≥0.4 [14].

### Statistics

Unpaired t-tests were used to compare, between Group A and Group B, the sensitivity, specificity, and agreement rates for both the entire recording period and each sleep stage. Paired t-tests were performed to compare sleep parameters determined from PSG and activity intensity data as well as sensitivity, specificity, and agreement rates obtained before and after the application of the optimization rules. Furthermore, ICC was calculated to analyze the correlation (agreement rate) between sleep parameters determined from PSG and activity intensity data. All data are expressed as mean ± SE. All statistical analysis was performed with IBM SPSS Statistics version 22.0. Statistical significance was set at *P* <0.05.

## Results

### Sleep/wake scoring algorithm

The S/W scoring algorithm below was obtained by performing discriminant analysis using the activity intensity and PSG data (total 4,080 epochs) obtained from the 17 participants in Group A.


Here, z ≥1 denotes wake (W_ACT_) and z <1 denotes sleep (S_ACT_). *x*_-2_, *x*_-1_, *x*, *x*_+1_, and *x*_+2_ indicate the activity intensity at 4 minutes before the evaluation epoch, at 2 minutes before, at the evaluation epoch, at 2 minutes after the evaluation epoch, and at 4 minutes after, respectively.

### Validity of the sleep/wake scoring algorithm

Table 1 shows the agreement rate, sensitivity, and specificity for each group between the S/W scoring algorithm using the activity intensity data and the S/W states determined visually from the PSG data. The agreement rates for the entire recording period for Group A and Group B were 84.7 ± 3.0% and 85.4 ± 2.8%, respectively, with no significant difference between the two groups (t(32) = -0.157, *P* =0.876). Similarly, there was no significant difference between the two agreement rates calculated for each sleep stage.

**Table 1:** **Accuracy of sleep/wake determination using the FS-750 actigraph**

		Group A ^a^	Group B ^a^	***t***	***p***
Agreement rates (%)	Overall	84.7 ± 3.0	85.4 ± 2.8	-0.157	0.876
	Stage W	66.0 ± 6.3	64.9 ± 7.1	0.114	0.910
	Stage 1	65.5 ± 6.5	65.2 ± 5.0	0.034	0.973
	Stage 2	91.5 ± 2.0	93.4 ± 1.2	-0.791	0.435
	Stage 3 + 4	99.0 ± 0.6	98.2 ± 0.7	0.884	0.383
	Stage REM	84.3 ± 5.3	82.4 ± 3.7	0.303	0.764
Sensitivity (%)		88.3 ± 2.8	90.0 ± 1.5	-0.522	0.605
Specificity (%)		66.0 ± 6.3	64.9 ± 7.1	0.114	0.910

^a^Values are expressed as mean ± SE.

Likewise, there was no significant difference in sensitivity and specificity of S/W determination between the groups. Therefore, equivalent score accuracies were obtained not only for the data in Group A, which were calculated using the S/W algorithm, but also for the data in Group B, which were independent sample sets.

### Optimizing the calculation of sleep parameters with the FS-750

Since there were no significant difference between the agreement rates for Group A and Group B for the entire recording period or each sleep stage, the data were analyzed by merging the data for both groups. Among the four sleep parameters, total sleep time (TST) and sleep efficiency (SE) are dependent on the values of sleep latency (SL) and wake after sleep onset (WASO), and therefore the optimization of SL_ACT_ and WASO_ACT_ was attempted.

**Figure 1:**
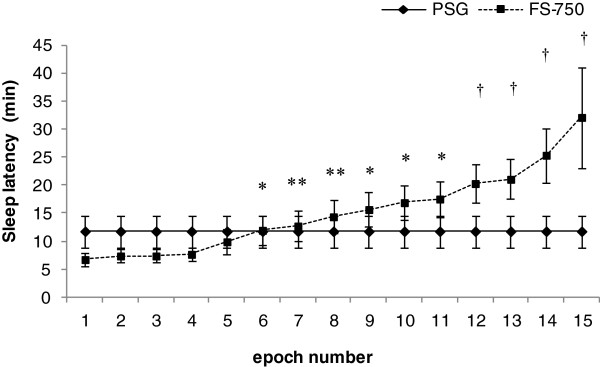
**Optimization of sleep latency determined by the FS-750 (SL**_**ACT**_ **)**_**.**_ SL_ACT_ is the interval between the time lights were turned off and the time of the first S_ACT_ (sleep-onset time) among the sleep states that appeared continuously for more than *n* epoch for the first time after the lights were turned off. The horizontal axis shows the *n* defined above. The vertical axis shows sleep latency (min) defined for each *n*. *P < .05 and **P < .01, significant intraclass correlation between sleep latency determined by polysomnography (SL_PSG_) and SL_ACT_. †*P* < .05, significant difference between SL_PSG_ and SL_ACT_ (paired t-test). Values are expressed as mean ± SE.

For the continuous number of epochs *n* =1 to 11 based on the definition of SL_ACT_ (see the Methods), there was no significant difference between SL_ACT_ and SL_PSG_. The difference was minimal when *n* was set to six epochs (Figure 1.) There was a significant intraclass correlation (ICC) between SL_ACT_ and SL_PSG_ when *n* was set between 6 and 11 (*P* <0.05), with ICC being ≥0.4 when *n* was 7 to 8. Therefore, the number of continuous epochs was chosen to be seven for SL_ACT_.

**Figure 2:**
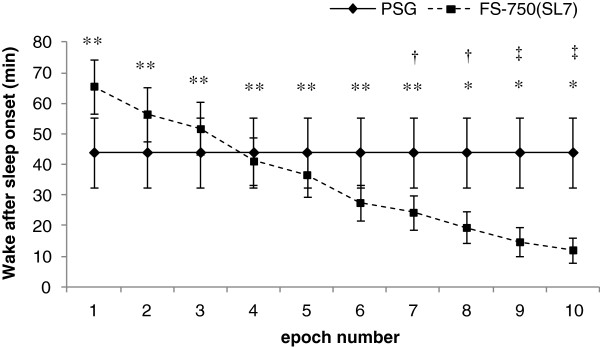
**Optimization of wake after sleep onset determined by the FS-750 (WASO**_**ACT**_ **).** WASO_ACT_ is the total time that wake epochs determined by the FS-750 (W_ACT_) appeared continuously for more than *n* epochs after sleep onset. The horizontal axis shows the *n* defined above. The vertical axis shows wake after sleep onset (min) defined for each *n*. *P < .05 and **P < .01, significant intraclass correlation between wake after sleep onset determined by polysomnography (WASO_PSG_) and WASO_ACT_. †*P* < .05 and ‡*P* < .01, significant difference between WASO_PSG_ and WASO_ACT_ (paired t-test). Values are expressed as mean ± SE.

The optimization of WASO_ACT_ was examined setting SL_ACT_ to seven epochs. In the range of one to six continuous epochs based on the definition of WASO_ACT_ (see the Methods), there was no significant difference between WASO_ACT_ and WASO_PSG_. The difference was minimal when *n* was set to four epochs (Figure 2). For a range between one and seven epochs, there was a significant intraclass correlation between WASO_ACT_ and WASO_PSG_ (*P* <0.01), with ICC being ≥0.4 in the corresponding range. As a result, the number of continuous epochs chosen for WASO_ACT_ was four.

The sleep parameters calculated by PSG and the activity intensity using the most optimal criteria (SL_ACT_: *n* =7 epochs, WASO_ACT_: *n* =4 epochs) are shown in Table 2. For each item, no significant difference was seen between the sleep parameters calculated using the PSG data and those calculated using the activity intensity, and there was a significant intraclass correlation between the two.

**Table 2:** **Optimized sleep parameters determined by the FS-750 actigraph**

			FS-750					
		Polysomnography						
			Before optimization			After optimization		
		Mean ± SE	Mean ± SE	***t***	ICC	Mean ± SE	***t***	ICC
Sleep latency	min	11.8 ± 2.9	6.7 ± 1.1	1.766	0.124	12.7 ± 2.7	-0.310	0.403**
Wake after sleep onset	min	44.1 ± 11.5	69.5 ± 8.8	-2.360*	0.419**	41.2 ± 7.9	0.271	0.427**
Total sleep time	min	424.2 ± 13.0	403.8 ± 8.7	1.716	0.411**	426.1 ± 8.0	-0.165	0.412**
Sleep efficiency	%	88.4 ± 2.7	84.1 ± 1.8	1.716	0.411**	88.8 ± 1.7	-0.166	0.412**

*P < .05, **P < .01.

Table 3 shows overall agreement rates, sensitivity (agreement rate for sleep state), and specificity (agreement rate for wakeful state) before and after the optimization of SL_ACT_ and WASO_ACT_. Despite a reduction in specificity between before optimization (SL_ACT_: *n* =1 epochs, WASO_ACT_: *n* =1 epochs) and after optimization (SL_ACT_: *n* =7 epochs, WASO_ACT_: *n* =4 epochs), overall agreement rates and sensitivity were improved significantly.

**Table 3:** **Overall agreement rates, sensitivity, and specificity for sleep/wake states before and after the application of optimization rules**

		Before optimization ^a^	After optimization ^a^	***t***	***p***
Agreement rates (%)	Overall	85.0 ± 2.0	88.4 ± 2.1	-6.087	<0.001
	Stage W	65.4 ± 4.7	56.5 ± 5.1	2.814	0.008
	Stage 1	65.4 ± 4.1	77.1 ± 3.9	-5.615	<0.001
	Stage 2	92.4 ± 1.2	96.2 ± 0.9	-7.159	<0.001
	Stage 3 + 4	98.6 ± 0.5	99.4 ± 0.4	-3.128	0.004
	Stage REM	83.3 ± 3.2	90.3 ± 2.8	-6.651	<0.001
Sensitivity (%)		89.1 ± 1.6	93.7 ± 1.4	-8.816	<0.001
Specificity (%)		65.4 ± 4.7	56.5 ± 5.1	2.814	0.008

^a^Values are expressed as mean ± SE (n =34).

## Discussion

It became clear that by using the S/W scoring algorithm developed in this study, the sleep/wake states of healthy adults could be determined from the FS-750 actigraph data with sensitivity and specificity equivalent to that determined with conventional actigraph data. Although the agreement rates in Stage Wake and Stage 1 were relatively low (about 65%), the agreement rates were high for stages 2 to 4 (≥90%). As a result, the overall agreement rate for the entire recording period was equivalent to that obtained using conventional actigraphs (about 85%) [1–6, 15]. The S/W scoring algorithm was developed in this study by using discriminant analysis that optimized differentiation between the sleep/wake states measured simultaneously by PSG. However, it should be pointed out that it would be difficult to determine the sleep states from the activity intensity data alone because mixtures of stages occur, such as when subjects are awake but motionless (silent awake) or during the early sleep-onset period when they are in light sleep and make some body movements. Accordingly, the agreement rate in Stage 1 or Stage Wake is generally lower compared to the agreement rate in other stages. According to a previous study [5], there is a tendency for the agreement rate to decrease during the light sleep stage, and the FS-750 shows a similar trend. The sleep/wake determination method thus appears to have acceptable determination accuracy given that the agreement rate for determining wakefulness by the FS-750 (specificity about 65%) is more than equivalent to that of conventional actigraphs [2–5, 15, 16].

The method was also optimized for calculating the sleep parameters SL, WASO, TST, and SE from the sleep/wake data for 2-minute epochs recorded by the FS-750. As a result, the values of these parameters could be predicted from the data obtained by the FS-750 and were very close to the values obtained by PSG (with no significant difference between them); there was also a significant intraclass correlation between the two. In addition, overall agreement rates were improved significantly after optimization. When looking at the values of the sleep parameters predicted by the FS-750 before the optimization (that is, when the number of continuous epochs for SL_ACT_ and WASO_ACT_ were defined as *n* =1), there was a tendency to underestimate SL and overestimate WASO (Figure 1, Table 2). By examining the criteria that minimized the differences between using PSG and activity intensity data, it was determined that SL_ACT_ ≥7 epochs (≥14 minutes of continuous sleep state determination) and WASO_ACT_ ≥4 epochs (≥8 minutes of continuous wake state determination) were optimal to apply to the estimation rule for sleep parameters. Large individual differences are apparent in the activity intensity data, as actigraphy tends to misjudge a subject who moves a lot while sleeping (restless sleep) as awake and, conversely, misjudges a subject who is awake but motionless (silent awake) as sleeping. In fact, application of the optimization rules improved sensitivity significantly. On the other hand, as this rule attaches importance to the duration, because a short wake state (<8 minutes) is misjudged as sleep, specificity is reduced. Specificity and sensitivity can be considered trade-offs, and as such the rules developed in this study are among the most appropriate evaluation indices for maximizing overall agreement rates.

There are some limitations to this study in that the evaluation epoch periods and the wearing positions of the device are different from those used in the previous studies and the sleep/wake patterns of restless sleep, silent awake, and short wake state are difficult to classify. Moreover, 30-second PSG data were re-scored as data for 2-minute epochs in this study to enable the algorithm to be developed. A longer epoch is more likely to contain different sleep/wake states and therefore tends to have lower agreement rates. Indeed, we forcibly converted data for each epoch determined by FS-750 (1 epoch =2 minutes) from 34 subjects into data for the corresponding four 30-second epochs, and compared the obtained results (30-second FS750 scores) with 30-second PSG scores. The overall agreement rate (85.0% versus 84.8%) and specificity (65.4% versus 59.9%) were significantly lower when comparing 30-second FS-750 scores and 30-second PSG scores than when comparing the corresponding 2-minute epoch score sets. On the other hand, there was no significant change in sensitivity (89.1% versus 89.2%). These results suggest that judgment errors related to the relatively low time resolution (2 minutes) need to be taken into consideration when using the FS-750 actigraph in clinical and research studies.

Additionally, the subjects of this study were healthy young adults. The relationship between sleep conditions and activity intensity might be different for elderly individuals who have difficulty achieving good quality sleep as well as for those with sleep disturbances [17]. As a next step, the accuracy of sleep/wake determination by the FS-750 needs to be verified in a larger sample and in various populations so that its utility can be determined. Its use by individuals with medical conditions and in other age groups should be further investigated. In the meantime, however, given that the FS-750 has a large memory that can store recordings over a long period, as well as capabilities to download the recordings at home, the device should help to push forward objective sleep evaluations for the elderly who find it difficult to visit healthcare facilities regularly, for individuals with circadian sleep disturbances who need longer term sleep studies, and for those who live in remote areas. As such, it is anticipated that the device will be utilized widely in both epidemiology studies and clinical practice.

## Conclusions

This study verified the accuracy of the sleep/wake states determined from the data obtained by the FS-750, an inexpensive device that has capabilities to download its data at home and which is a promising tool to use in large epidemiological studies and in remote sleep medicine. In addition, the algorithm developed can determine the sleep/wake states in 2-minute epochs using the activity intensity data measured by the FS-750. The agreement rate between the results calculated with the FS-750 and those calculated with PSG was high (85%), and the sensitivity and specificity of measurement using the FS-750 were equivalent to those determined with conventional actigraphs. The device’s application to future sleep research and sleep medicine is expected.

## Abbreviations

AHI: apnea and hypopnea index
ECG: electrocardiogram
EEG: electroencephalogram
EMG: electromyogram
EOG: electrooculogram
NFC: near field communication
PLMI: periodic limb movement index
PSG: polysomnography
RH: relative humidity
S_ACT_: sleep epochs determined by the FS-750
SE: sleep efficiency
SL: sleep latency
SL_ACT_: sleep latency determined by the FS-750
SL_PSG_: sleep latency determined by PSG
S_PSG_: sleep epochs determined by PSG
S/W: sleep/wake
TIB: time in bed
TST: total sleep time
W_ACT_: wake epochs determined by the FS-750
WASO: wake after sleep onset
WASO_ACT_: wake after sleep onset determined by the FS-750
WASO_PSG_: wake after sleep onset determined by PSG
W_PSG_: wake epochs determined by PSG.
